# Structural and biological characterization of shortened derivatives of the cathelicidin PMAP-36

**DOI:** 10.1038/s41598-023-41945-1

**Published:** 2023-09-13

**Authors:** Barbara Biondi, Luigi de Pascale, Mario Mardirossian, Adriana Di Stasi, Matteo Favaro, Marco Scocchi, Cristina Peggion

**Affiliations:** 1 Institute of Biomolecular Chemistry, CNR, Padova Unit, Padova, Italy; 2 Department of Life Sciences, University of Trieste, Trieste, Italy; 3 Department of Chemical Sciences, University of Padova, Padova, Italy

**Keywords:** Structure elucidation, Peptides

## Abstract

Cathelicidins, a family of host defence peptides in vertebrates, play an important role in the innate immune response, exhibiting antimicrobial activity against many bacteria, as well as viruses and fungi. This work describes the design and synthesis of shortened analogues of porcine cathelicidin PMAP-36, which contain structural changes to improve the pharmacokinetic properties. In particular, 20-mers based on PMAP-36 (residues 12-31) and 13-mers (residues 12-24) with modification of amino acid residues at critical positions and introduction of lipid moieties of different lengths were studied to identify the physical parameters, including hydrophobicity, charge, and helical structure, required to optimise their antibacterial activity. Extensive conformational analysis, performed by CD and NMR, revealed that the substitution of Pro25-Pro26 with Ala25-Lys26 increased the α-helix content of the 20-mer peptides, resulting in broad-spectrum antibacterial activity against *Escherichia coli, Staphylococcus aureus, Klebsiella pneumoniae, Acinetobacter baumannii, Pseudomonas aeruginosa and Staphylococcus epidermidis* strains. Interestingly, shortening to just 13 residues resulted in only a slight decrease in antibacterial activity. Furthermore, two sequences, a 13-mer and a 20-mer, did not show cytotoxicity against HaCat cells up to 64 µM, indicating that both derivatives are not only effective but also selective antimicrobial peptides. In the short peptide, the introduction of the helicogenic α-aminoisobutyric acid forced the helix toward a prevailing 3_10_ structure, allowing the antimicrobial activity to be maintained. Preliminary tests of resistance to Ser protease chymotrypsin indicated that this modification resulted in a peptide with an increased in vivo lifespan. Thus, some of the PMAP-36 derivatives studied in this work show a good balance between chain length, antibacterial activity, and selectivity, so they represent a good starting point for the development of even more effective and proteolysis-resistant active peptides.

## Introduction

Antimicrobial resistance (AMR) is a serious threat to human health worldwide. Therefore, the interest in new drug candidates with antimicrobial activity, as new agents that could substitute or complement conventional antibiotics, is steadily increasing^1^. Antimicrobial peptides (AMPs) are an ancient class of molecules that form a first line of defence in eukaryotes to combat microorganisms. Attention on this class of compounds has risen because their most common mechanism of action targets bacterial membranes or other general structures, in a manner different to most antibiotics, that typically target specific proteins or membrane components^2^. This specific mode of action makes the development of bacterial resistance less likely to occur, thus making AMPs promising antibacterial drugs^3^. Thousands of AMPs have now been isolated from a variety of natural sources comprising microorganisms, plants, and animals including mammals^4^. The vast pool of natural antimicrobial peptides offers a broad spectrum of biological activities to choose from, but often these compounds are not stable, with a short half-life, and when used out of their innate immune context are often quite toxic to host cells. To allow for clinical applications, it would be important to develop new artificial, optimised and long lived AMP analogues that overcome the drawbacks of the natural peptides^5^.

Cathelicidins are a family of antimicrobial peptides in vertebrates that plays an important role in the innate immune response^6, 7^. They contain highly variable C-terminal domains displaying direct antimicrobial activity against a variety of bacteria, fungi, and some virus and parasites^8^. Porcine myeloid PMAP-36 is a highly cationic, 36-amino acid long cathelicidin displaying antimicrobial activity (in vitro and in vivo)^9, 10^ as well as immunomodulatory properties^11, 12^. The N-terminal region is characterised by several charged residues and is organised as an α-helical structure, interrupted by three proline residues (Pro25, Pro26 and Pro32) located at the C-terminal end^12, 13^. Helicity was predicted by helical wheel projections and then verified by circular dichroism measurements in phosphate buffer pH 7, with addition of 25% TFE. The presence of a cysteine (Cys35) determines the formation of peptide homodimers with a total net charge of + 26. Dimerization probably occurs in storage form of the peptide’s precursors in peripheral white blood cells^13^.

The peptide displayed a broad spectrum of antibacterial activity against Gram+ and Gram− bacteria at µM concentrations and against *Candida* as well^10, 12–15^, but it also exhibited a significant cytotoxicity which is likely associated to its membranolytic activity^13–16^.

Structure–activity studies have been performed to improve the antimicrobial activity and reduce the cytotoxic effects of this peptide. Monomerization did not affect the antibacterial properties of PMAP-36^10, 12–15^. On the other hand, the N-terminal segment 1-24 fully retained a comparable antimicrobial activity to the entire peptide and increased its selectivity for prokaryotic cells by showing lower haemolytic activity^15^, while the shorter fragment 1-20 exhibited decreased potency^13^. Further truncation of the first six N-terminal residues of the peptide 1-24 to obtain the 18-mer peptide encompassing residues 7-24 of the native peptide instead resulted in a fully active peptide, suggesting that hydrophobic residues upstream of the two prolines are more important for activity than the six N-terminal residues^14^. In addition, Scheenstra et al. showed that the N-terminal truncation of 11 residues in the PMAP-36 monomer had little effect on killing of *E. coli*, while further truncation of the peptide completely blocked its antibacterial activity^12^. All these data are consistent with the indication that the central part of the molecules is the pharmacophore.

In this work, we designed and tested a series of shortened PMAP-36 analogues focusing on the 12-31 and 12-24 regions, which constitute the central and most active portion of PMAP-36.

The aim was to clarify the importance of the α-helical structure and to find the most active while least cytotoxic derivatives. The structures of these new analogues were analysed by CD and NMR and their antibacterial effects, cytotoxicity and stability were studied. The results provide interesting new data for further optimization of these molecules for future use as therapeutics.

## Results

### Design, synthesis, and physicochemical parameters of PMAP-36 derivatives

The peptides designed for this study are shortened analogues of PMAP-36, lacking the dispensable N-terminal portion 1-11 and covering the region between Lys12 and Ile31. The C-terminal portion 32-36 of the original PMAP-36 was also removed to prevent kinking of the helical structure due to Pro32 and to avoid dimerization caused by Cys35.

**Figure 1:**
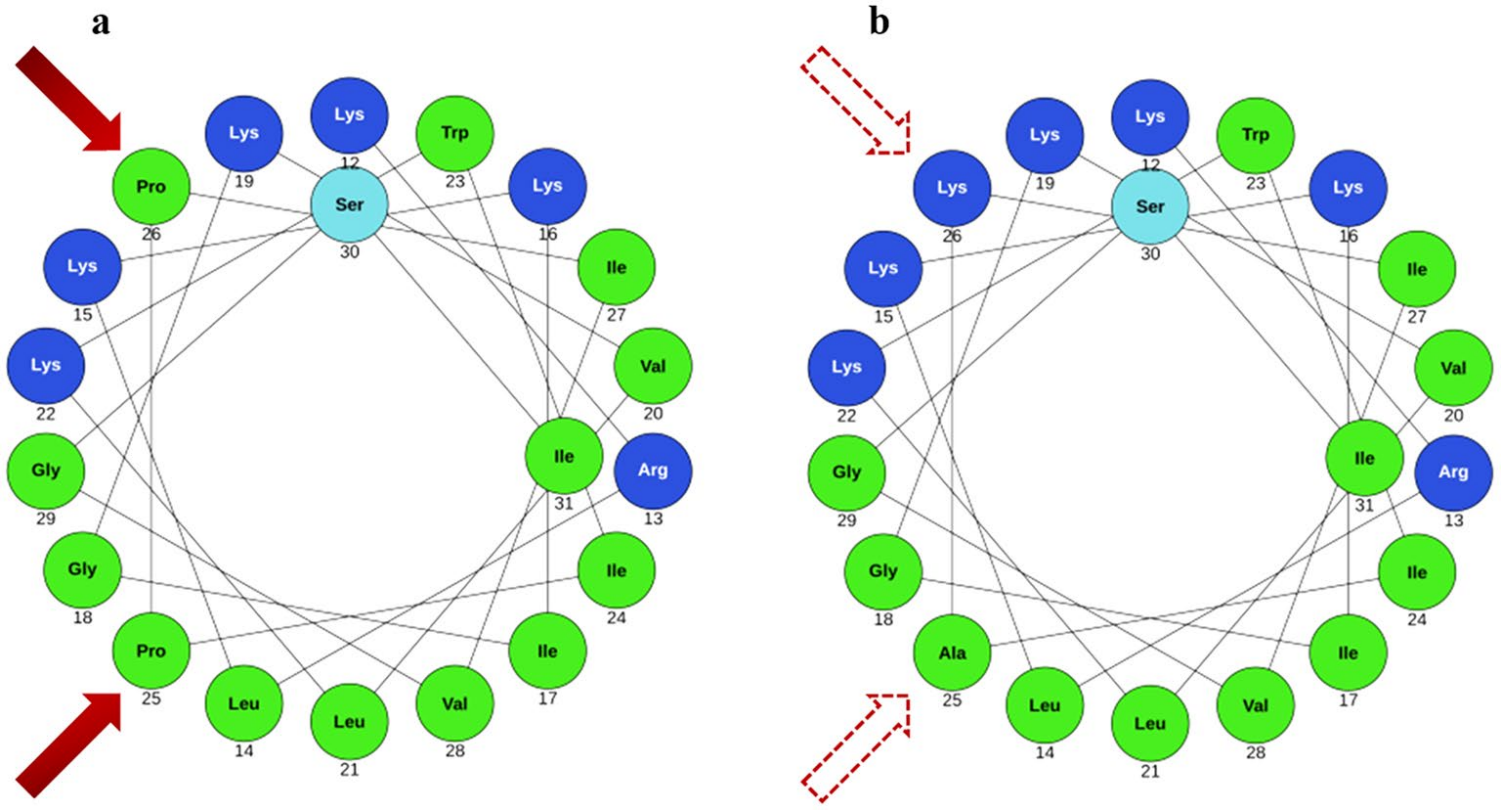
Helical wheel projections of [P25,P26]-PMAP12-31, compound **a**, (*left*) and [A25,K26]-PMAP12-31, compound **b** (*right*). Cationic residues are in blue, hydrophobic residues in green. (https://www.donarmstrong.com/cgi-bin/wheel.pl).

The remaining 20-mer (12-31) (compound **a**, Table 1) contains two prolines that interrupt the helical structure (Fig. 1). To evaluate the effects of extending the α-helical conformation to the entire sequence, a new compound was designed in which Pro25 was replaced by Ala25 and Pro26 by Lys26 (compound **b**, Table 1), to also increase the charge, which is important for antimicrobial activity. The hydrophobic and the cationic residues were inserted in a manner that would increase the amphiphilic character of the resulting α-helix, as perceived in the helical wheel projections (Fig. 1).

**Table 1:** List of PMAP-36 analogues and of their physico-chemical properties.

	Peptide sequence	Theoretical MW	Experimental MW	Retention time^a^	Hydrophobicity^b^	Helicity (%)^c^
		[M + H]^+^_calcd_	[M + H]^+^_exp_	t_R_ (min)	Δt_R_	
**PMAP-36**	*KRLKKIGKVLKWIPPIVGSIPLGCG-NH_2_					
**a**	Ac-KRLKKIGKVLKWIPPIVGSI-NH_2_	2314.5	2314.8	13.2	0	18
**b**	Ac-KRLKKIGKVLKWI**AK**IVGSI-NH_2_	2319.5	2319.7	17.2	4.0	94
**c**	Oct-KRLKKIGKVLKWI**AK**IVGSI-NH_2_	2404.2	2403.8	22.3	9.1	71
**d**	Lau-KRLKKIGKVLKWI**AK**IVGSI-NH_2_	2460.3	2459.7	22.8	9.6	47
**e**	Pal-KRLKKIGKVLKWI**AK**IVGSI-NH_2_	2516.4	2516.9	25.8	12.6	53
**f**	Ac-KRLKKIGKVLKWI**AK**IVGSI-**Aun**-NH_2_	2503.3	2503.8	20.3	7.1	82
**g**	Ac-KRLKKIGKVLKWI-NH_2_	1650.2	1650.5	12.1	− 1.1	ND^d^
**h**	Ac-KRLKKIGKV**U**KWI-NH_2_	1623.1	1623.4	11.7	− 1.5	ND

*Omitted N-terminal sequence = GRFRRLRKKTR. ^a ^Retention time refers to 5%—95%B (CH_3_CN/H_2_O, 9:1) over 30 min gradient, C18 column; relative to compound **a**: ^b^Δt_R_ = t_R_(**x**)−t_R_(**a**), where **x** stays for peptides **b**–**h**; ^c^calculated from the [θ]_222_ value in SDS solution^17^; ^d^ND, not determined for mixed helices. Aun: 11-aminoundecanoic acid; Aib, U: α-aminoisobutyric acid.

Furthermore, we prepared a series of analogues based on compound **b** ([A25,K26]-PMAP12-31) modified by linking an acyl chain of different length. Analogues **c**, **d**, and **e** were prepared by respectively linking octanoic, lauric, and palmitic acid to the N-terminus of the sequence. Thus, we investigated whether variations in the lipid moiety could modulate the insertion of the derivatives into bacterial membranes, and if it could also influence their antimicrobial potency. To investigate if the position of the lipid chain impacts the biologic effects, 11-aminoundecanoic acid (Aun) was linked to the C-terminus of compound **b** giving the analogue **f**. This analogue was intended to be compared with analogue **d** to evaluate the different effects of lipidation at the N- and C-termini.

To exclude the possibility that any difference between lipidated and non lipidated peptides might be due to the lack of the free positively charged N-terminal amino group, all non-lipidated analogues were acetylated at their N-terminus. In this way the number of amides in peptides of the same length remained unmodified. Furthermore, all peptides were amidated at their C-terminus as is the natural PMAP-36^13^.

A shorter analogue with only 13 amino acids (derivative **g**) was also designed to test whether a further shortening of the 20-mer derivative (**a**) could produce a compound capable of maintaining antibacterial activity. Since peptide shortening often leads to a decrease in helix stability, derivative **h** was also prepared, in which Leu21 was replaced by α-aminoisobutyric acid (Aib, U). This non-proteinogenic amino acid is known for its strong ability to promote helix folding in synthetic and natural sequences^18–20^. The complete series of PMAP-36 analogues is described in Table 1.

### Conformational analysis of PMAP-36 derivatives

The secondary structure of the peptides was investigated by circular dichroism (CD) spectroscopy in various environments, including 0.2 mM potassium-phosphate buffer (aqueous solution, pH 7), 100 mM sodium dodecyl sulphate (SDS), and 2,2,2-trifluoroethanol (TFE). As these peptides are active on membranes, we chose SDS, as SDS micelles represent a model mimicking the prokaryotic cell membrane. TFE, on the other hand, is a solvent that induces the formation of helical structures^21^.

**Figure 2:**
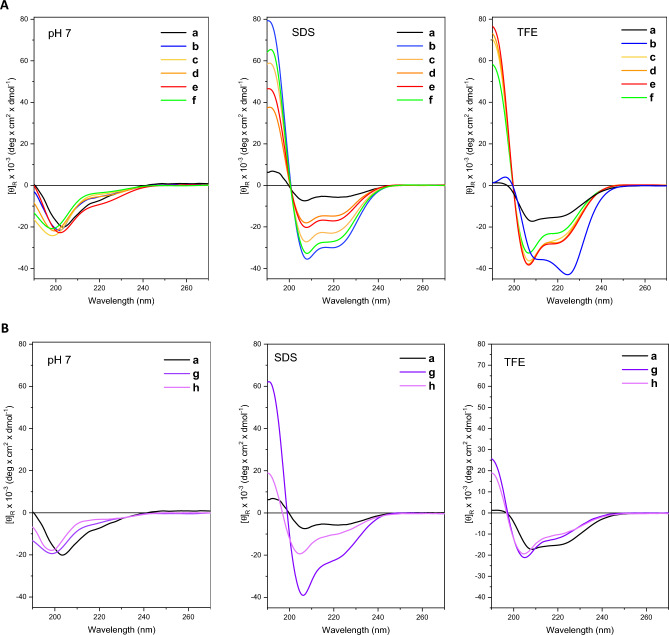
(**A**) CD spectra of PMAP12-31 **a**–**f** analogues in PBS, SDS and TFE, at 25 °C. (**B**) CD spectra of peptides **a**, **g** and **h** in PBS, SDS and TFE, at 25 °C.

All analogues showed an unordered conformation in aqueous solution with a negative peak in the region around 200 nm (Fig. 2A).

Peptide **a** showed a modest helical structure (18% helicity) in the presence of SDS micelles, which are a first approximation of the amphiphilic environment of biological membranes, while the substitution of Pro25 and Pro26, respectively, by Ala and Lys, significantly increased the helical content (94% helicity) (Fig. 2A). The CD spectrum of peptides **b–f** in SDS showed the typical three-band shape with two negative maxima at 208 and 222 nm, assigned to the n → π* transition and to the parallel component of the π→π* transition respectively, and a positive maximum around 195 nm attributed to the antiparallel component of the π→π* transition (Fig. 2A). The presence of an acyl chain of different lengths at the N-terminus in the analogues **c**, **d,** and **e** resulted in a perturbation of the helical structure in the form of a decrease in helicity. In contrast, the introduction of Aun at the C-terminus (peptide **f**) did not significantly affect the conformation of the peptide (Fig. 2A). A similar behaviour was observed for the analogues **c**–**f** analysed in TFE, condition that however evidenced an anomalous CD spectrum for peptide **b** (Fig. 2A), in which the observed ratio between molar ellipticities at 222 and 208 nm was > 1, suggesting an aggregation phenomenon compatible with the presence of a coiled-coil structure^16, 22^.

The short peptides **g** and **h** showed an ordered conformation in SDS and TFE (Fig. 2B). CD spectra deviated from those of a pure α-helix in both peptides. We observed a shift of one of the two negative maxima to around 205 nm, accompanied by a shoulder centred near 222 nm. Furthermore, the value of the ratio *R*([Θ]_222_/[Θ]_204–206_) moves away from the ideal 1 of the α-helix with values around 0.4–0.5 typical of the 3_10_-helix, values that were first theorised^23^ and then determined in the first unequivocal experimental CD spectrum reported for an ideal right-handed 3_10_-helical peptide^24, 25^. The contribution of the 3_10_-helix conformation was different for peptides **g** and **h**, and in the two different membrane mimetic environments studied.

CD spectra of peptide **g** in SDS showed a contribution, albeit modest, of the 3_10_-helix, as evidenced by the *R*([Θ]_222_/[Θ]_204–206_) value of 0.55 and by the shift of the negative maxima. At the same time, there was still a relevant contribution of α-helix as evidenced by the very intense positive band at 195 nm. The contribution of 3_10_-helix appeared to be more significant in peptide **h**. Furthermore, to a *R* value of 0.55, the ellipticity at about 195 nm was only slightly positive in the case of peptide **h**. Both **g** and **h** showed low ellipticity at 195 nm in TFE and *R* ratios of 0.55 and 0.58, respectively. These results could be due to an equilibrium of 3_10_- and α-helices and/or from 3_10_ and α-helix segments that coexist in the same molecule.

We performed a 2D-NMR analysis in TFE-*d*2 solution to obtain a more in-depth view of the conformational preference of some of the analogues, focussing on peptides** a**, **b**, **g,** and **h**.

The NMR spectra of **a** evidenced little dispersion of the proton resonances and extensive overlapping, suggesting poor organisation in helical structure (data not shown).

In contrast, peptides **b, g,** and **h** exhibited greater signal dispersion, indicating well-organised structures. Complete assignment of proton resonances was established based on TOCSY and NOESY spectra, using the Wüthrich’s procedure^26^ (Table S1–S3 and Figs. S1–S5 in Supporting Information).

In general, helical structures are readily identified by a series of consecutive NN(i, i + 1) NOEs, concomitant with αN(i, i + 3) NOEs^26–28^. These connectivities, evident for **b**, **g** and **h**, reveal short interproton distances (< 3.5 Å) that demonstrate helical structure, without distinguishing between the different helix types^27^.

3_10_-helix and α-helix are distinguished by the relative intensities of the intermediate range αN(i, i + 2) and αN(i, i + 4) NOEs. It is known that 3_10_ -helices should display αN(i, i + 2) and αN(i, i + 3), whereas α-helices display only αN(i, i + 3) and αN(i, i + 4) NOEs^26, 27, 29^.

**Figure 3:**
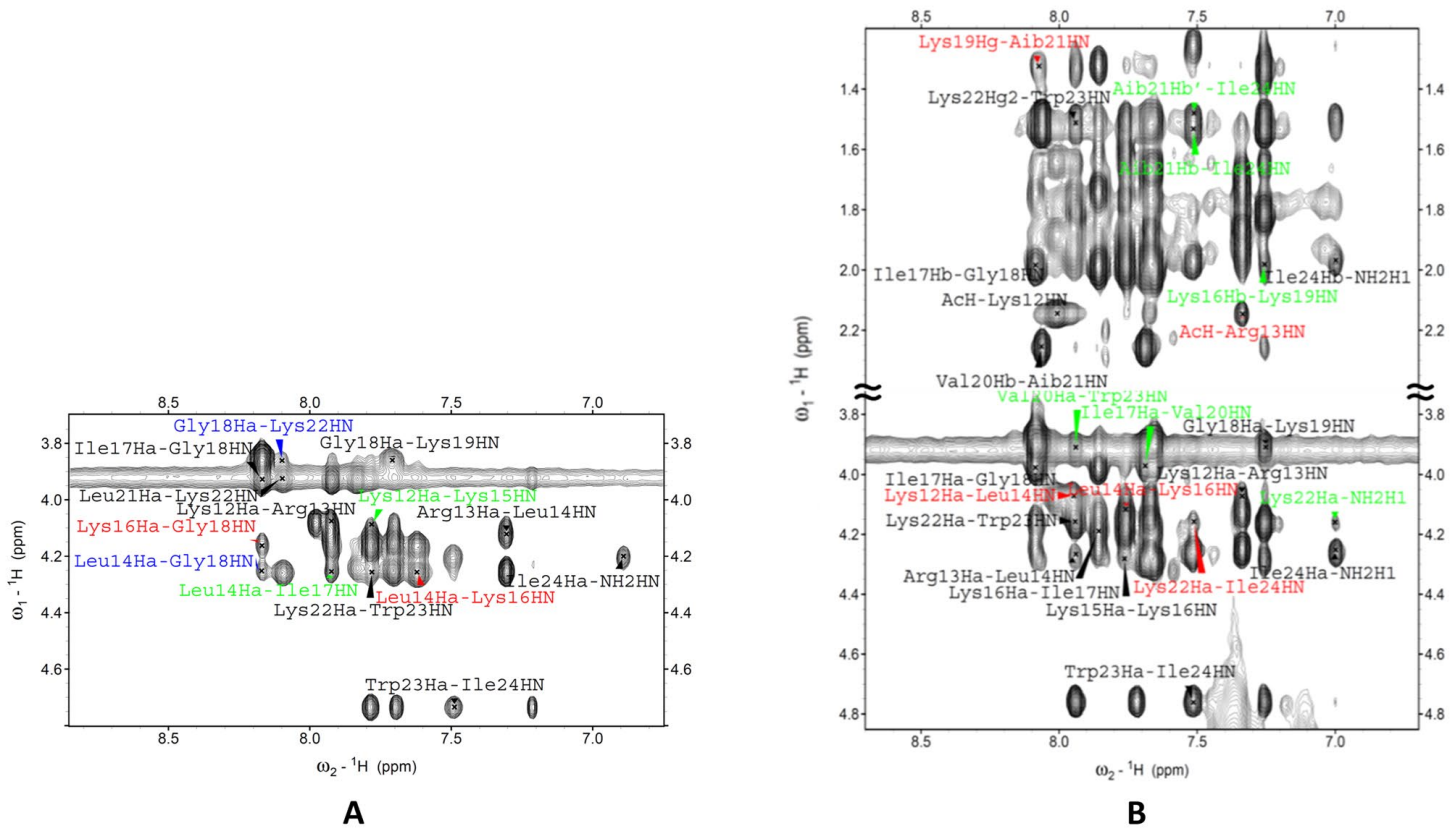
(**A**) Fingerprint region of the NOESY spectrum (600 MHz) of peptide **g** in TFE solution (c = 1.2 mM, T = 308 K). αN(i, i + 2), αN(i, i + 3), and αN(i, i + 4), cross-peaks are highlighted in red, green, and blue, respectively. (**B**) Regions of the NOESY spectrum (600 MHz) of peptide **h** in TFE solution (c = 1.2  mM, T = 308 K). α/βN(i, i + 2) and α/βN(i, i + 3) cross-peaks are highlighted in red, and green, respectively.

In Fig. 3A the fingerprint region of the NOESY spectrum of peptide **g** is reported. Together with all sequential cross peaks αN(i, i + 1), we observed two αN(i, i + 2), Leu14/Lys16 and Lys16/Gly18, several long range correlations involving αN(i, i + 3)_*,*_ Lys12/Lys15 and Leu14/Ile17, and αN(i, i + 4), Leu14/Gly18 and Gly18/Leu22. These findings confirmed the adoption of a mixed 3_10_-/ɑ-helical conformation for **g**.

Figure 3B shows regions of the NOESY spectrum of **h**. In the fingerprint region we observed all sequential cross peaks αN(i, i + 1), three αN(i, i + 2), Lys12/Leu14, Leu14/Lys16 and Lys22/Ile24, and several intermediate-range correlations involving αN(i, i + 3)_*,*_ Ile17/Val20, Val20/Trp23 and Lys22/NH(C-terminus amide). The βN(i, i + 3) signals involving Aib21 are observed in the aliphatic region. The absence of the long range α/βN(i, i + 4) correlations is indicative of a more pronounced 3_10_-helical character for peptide **h**, with respect to peptide **g**.

### Antibacterial and cytotoxicity of PMAP-36 derivatives

All derivatives were tested on a representative panel of reference strains of Gram-positive and Gram-negative bacteria (Table 2). For this purpose, the minimum concentration needed to inhibit bacterial growth (MIC) was measured. Peptide **a** effectively inhibited all tested bacterial strains (MIC = 1–4 µM) with the sole exception of *S. aureus* (MIC = 64 µM). The substitution of the two prolines in peptide **b** markedly changed the spectrum of activity, making the peptide active against *S. aureus* but, at the same time, decreasing its antimicrobial activity against all other bacterial species by 2–8 times (Table 2).

**Table 2:**
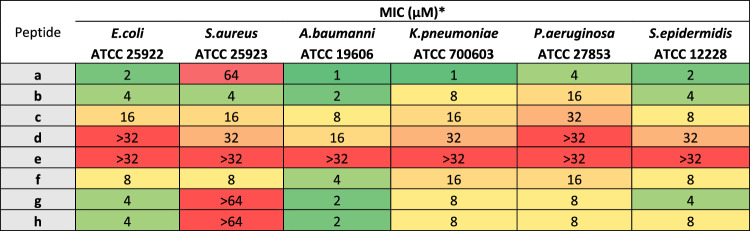
Antibacterial activity (MIC) of PMAP-36 derivatives against Gram-negative and Gram-positive bacteria.

*MIC, minimum inhibitory concentration is the lowest peptide concentration causing no visible growth after 18 h incubation in Mueller–Hinton broth at 37 °C. Values indicate the mode of three independent tests. Coloured boxes indicate different antimicrobial activity: high activity (green) low or no activity (red). Lipopeptides have not been tested above 32 µM due to their low solubility (see *Supporting Information* for evaluation of aggregation).

Lipopeptides **c**, **d**, and **e** generally decreased their antibacterial potency proportionally to the length of the carbon chain. Interestingly, lipopeptide **f** with Aun at the C-terminus showed better activity than compound **d,** bearing a lipid tail of the same length but at its N-terminus. The overall results with these lipopeptides suggest that the addition of a lipid moiety to the peptides at the C-terminus of PMAP12-31 is less detrimental than at its N-terminus, but does not improve antibacterial activity. The low solubility of compounds **c**, **d** and **e** in Müller-Hinton broth (see Supporting Information Fig. S6) also limited the MIC assay to peptide concentrations below 64 µM and may have contributed to the scarce antimicrobial activity of these compounds.

Interestingly, the shorter derivatives **g** and **h** still resulted in active peptides with a comparable activity spectrum to that of peptide **a** and only a slightly reduced activity (MIC = 2–8 µM, except against *S. aureus* MIC > 64 µM). The substitution of Leu21 with Aib in peptide **h** did not substantially modify its antimicrobial activity compared to peptide **g** (Table 2).

**Figure 4:**
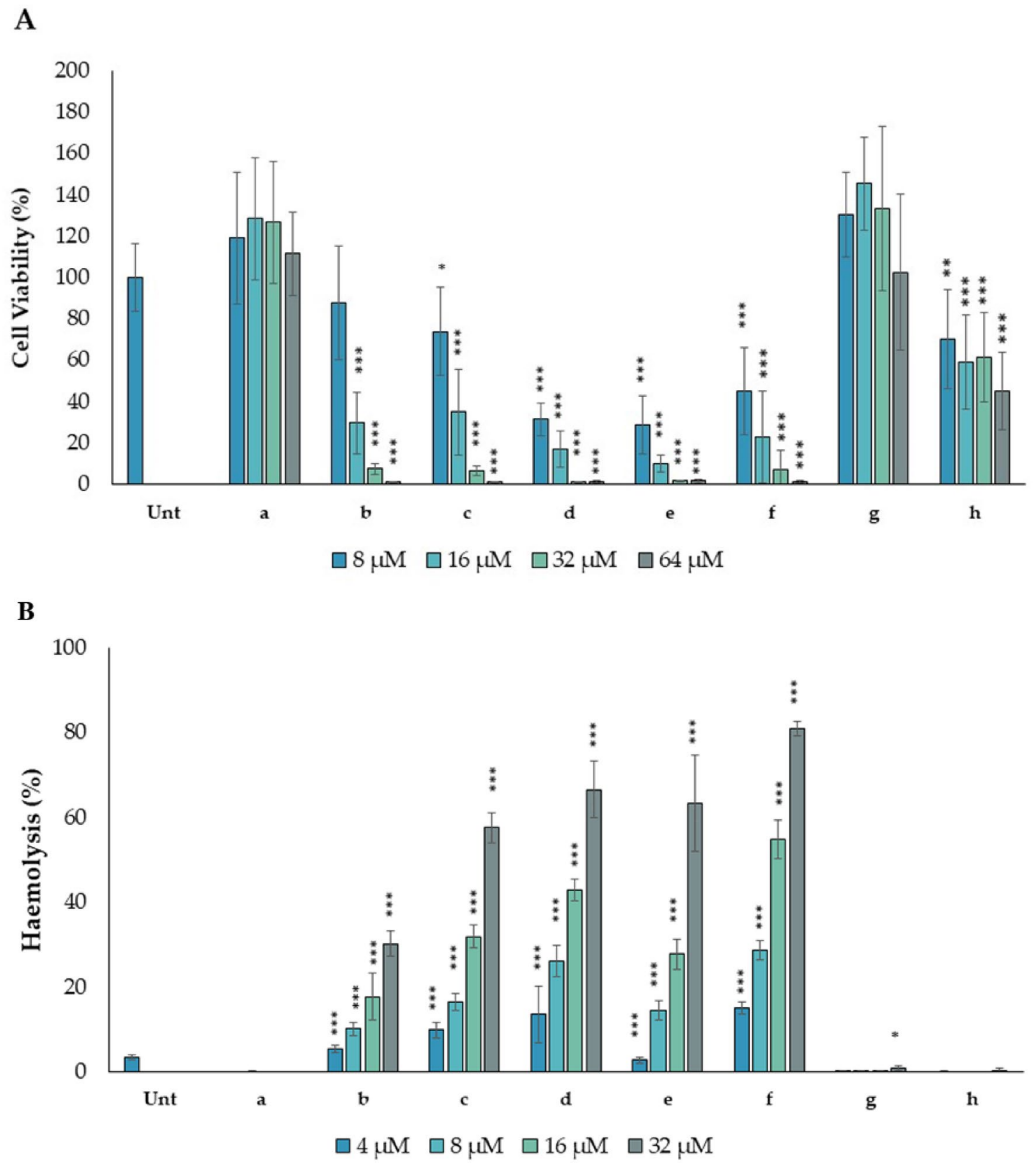
Effects of PMAP-36 derivatives on the viability of the HaCaT cell line (**A**) and red blood cells (**B**). (**A**) Cell viability was measured as absorbance at 570 nm after 24 h from the exposure to the peptides. Results are reported as percentages of viable cells with respect to the untreated control cells (Unt) set as 100% of viability. Data are the average ± SD of at least three independent experiments in internal duplicate (n = 6). *p < 0.05, **p < 0.01, ***p < 0.001 versus the untreated control (Test t-student). (**B**) Haemolysis assay was performed against 4% v/v suspension of ram red blood cells (RBCs) in PBS. Absorbance of released haemoglobin (540 nm) were measured after 1 h exposure to the peptides. RBCs suspension with 1% Triton or without peptide treatment were used to achieve 100% haemolysis and as negative haemolysis control (Unt), respectively. The results are shown as a percentage with respect to of RBCs treated for 1 h with 1% Triton X-100. Data are the average ± SD of three experiments (n = 3). *p < 0.05, ** p < 0.01, *** p < 0.001 versus the untreated sample (Test t-student).

The effects of PMAP-36 derivatives were also observed in eukaryotic cells. To determine the viability of the cells in the presence of the peptides, the metabolic MTT assay was performed using the HaCaT cell line incubated with different concentrations of each compound (Fig. 4A). Peptides **a** and **g** did not decrease cell viability even at 64 µM after 24 h of incubation. On the contrary, peptide **b** affected cell viability, which dropped to 30% (compared to an untreated control) at 16 µM and even further at higher concentrations of the compounds. Lipopeptides **c, d, e,** and **f** showed significant cytotoxicity in the same range of concentrations required for antimicrobial activity (8–16 µM), with HC50 values < 32 μM. Surprisingly, peptide **h** containing Aib, decreased cell viability at a concentration of 8 µM to just 70% (compared to an untreated control) and is at or above the 50% viability value up to 32 μM. To determine whether the mechanism of cytotoxicity was due to membrane disruption, a haemolysis assay was performed on red blood cells (RBCs). It was confirmed that peptides **a**, **g**, and **h** had no membranolytic effect on erythrocytes (Fig. 4B). On the other hand, peptides **b**, and lipopeptides **c**, **d**, **e**, and **f** showed a significant lytic effect at all concentrations tested, suggesting that the cytotoxicity observed in eukaryotic cells were mainly due to membrane damage. Interestingly, peptide **h**, albeit slightly metabolically cytotoxic to HaCaT cells, did not appear to damage erythrocytes (Fig. 4B). Therefore, further studies will be needed to understand the mechanism of cytotoxicity of peptide **h** on the HaCaT cells.

#### Resistance to protease

**Figure 5:**
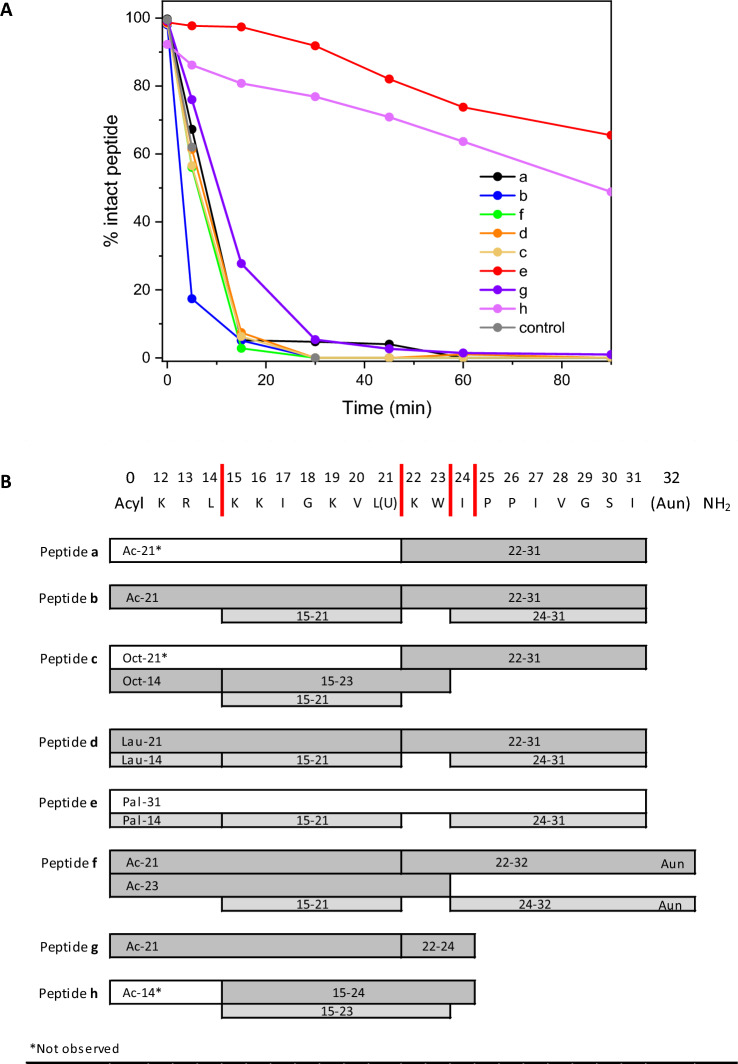
(**A**) Chymotrypsin degradation of compounds **a**–**h**. The percentage of preserved peptide is represented over time. We estimated the residual amount of intact peptide in a sample as the HPLC peak area. (An example of the HPLC profiles with labelled peaks corresponding to hydrolysis products is reported in the Supporting Information, Fig. S8). As a positive control we used the C-terminal portion of natural Endonuclease V involved in the DNA repair mechanism that occurs after DNA damage caused by ultraviolet radiation. (**B**). General sequences of peptides **a**–**h** and their degradation products by chymotrypsin. All peptide fragments were detected by HPLC–MS (see Materials and Methods). Fragments obtained within a 30-min incubation with chymotrypsin are indicated by dark grey colour; fragments obtained after longer incubation times are indicated by light grey colour. White boxes indicate hypothetical fragments not detected. Vertical red bars in the general sequence (top) indicate the position of observed cleavage sites.

The stability of the peptides in the presence of chymotrypsin was monitored by HPLC–MS, analysing the fragments derived from the degradation of the peptide. All 20-mer analogues (**a**–**f**) are sensitive to chymotrypsin. The rate of peptide degradation was calculated as the percentage of intact peptide detected at defined time points. All peptides were mostly degraded within the first 15 min of incubation, with the exception of the lipopeptide **e** and the short peptide **h**. After 90 min, 65% of the peptide **e** was still intact, indicating that palmitoylation protects against hydrolysis. For peptide **h**, which was designed with an Aib residue to improve proteolytic resistance, 50% of the peptide was still intact after 90 min (Fig. 5A). The other peptides were cleaved on the C-terminal side of Leu21 or Trp23 as the first cleavage observed. Successive degradations involve Leu14 for all peptides (with the exception of **a** and **h**). Concerning 13-mer peptides (**g** and **h**), degradation at position 21 occurred only for **g** and not for the Aib containing peptide **h**, for which Leu14 is the sensitive point for chymotrypsin attack. A summary of the fragments detected for each peptide is shown in Fig. 5B.

### Discussion

It is well known that the transition of AMP use from the laboratory to real applications is hampered by their poor pharmacokinetic properties. An effective way to overcome this weakness of AMPs is to introduce structural changes that increase resistance to proteases, while maintaining or improving activity and, at the same time limiting toxicity.

In this study, we synthesised and tested a series of PMAP-36 derivatives by shortening the sequence length, changing amino acid residues at critical positions, and inserting lipid moieties. This helped to identify physical parameters, including hydrophobicity, charge, and helicity, required to optimise the antibacterial activity and selectivity of this peptide.

We first showed that the shortened PMAP12-31 peptide **a**, which is the monomeric central part of the natural PMAP-36, exhibited extensive broad-spectrum antibacterial activity against *E. coli*, *A. baumannii, K. pneumoniae, P. aeruginosa*, and *S. epidermidis* strains with MIC values of 1–4 µM. Interestingly, we found that this peptide could be further shortened to 13-mer 12-24 (compound **g**) with only a 1–8-fold decrease in antibacterial potency, depending on the pathogen. Interestingly, both compounds **a** and **g** were not cytotoxic against HaCaT cells up to 64 µM and not haemolytic, indicating that both derivatives are effective and selective antimicrobial compounds (see Table 3 and Figs. S9, S10 in Supporting Information) and useful leads for further development. In addition, *A. baumannii* and *K. pneumoniae,* which are among the most worrying pathogens due to their high degree of resistance^30^, and have never been tested against PMAP-36 derivatives, were found to be highly sensitive to both compounds **a** and **g**. Peptide **g** is almost identical to the 12-mer peptide RI12 reported in Lyu et al.^14^ and Lv et al.^15^, however, it contains an additional acetylated lysine at the N-terminus. Both peptides are not haemolytic and are active against Gram-negative but are only scarcely active against Gram positive bacteria. The addition of a charged residue in **g** increased the antimicrobial activity against the *S. epidermidis* strain.

**Table 3:** Selective antibacterial activity of PMAP-36 derivatives.

Peptide	Minimum selectivity index*	
	IC_50_ /MIC	MHC/MIC
**a**	> 16	> 8
**b**	≥ 1.6	≥ 1
**c**	≤ 1.6	< 0.5
**d**	< 0.2	≤ 0.1
**e**	< 0.2	< 0.2
**f**	< 1	< 0.5
**g**	> 8	> 4
**h**	≥ 6.8	> 4

*Minimum selectivity index is calculated as ratio between cytotoxicity/haemolytic activity and MIC on at least five out of six bacterial species. The IC_50_ was considered as the peptide concentration at which cell viability is reduced by 50% compared to the untreated control. MHC was taken as the lowest concentration of peptides which induced 10% of haemolysis of red blood cells^31^.

Pro25 and Pro26 of peptide **a** were replaced by the two helical promoting residues Ala and Lys (compound **b**) attempting to increase the amphipatic α-helix content of the peptide. CD analysis and NOESY spectrum confirmed the significant increase in peptide structuration with 94% α-helical conformation in the presence of membrane-mimetic environments. In addition, compound **b** displayed hydrophobicity higher than that of peptide **a** likely due to the enhanced amphipathicity of the helix, acquiring biological activity against *S. aureus* (MIC 4 µM), but at the same time decreasing activity 2–8-fold against the other strains. Furthermore, this compound also showed an increase in cytotoxicity compared to compound **a**. Because compounds **a** and **b** have a similar charge, the main differences are the increased helicity and hydrophobicity of **b,** which did not result in a more selective peptide (Table 3). These results are consistent with those of other studies^32, 33^ in which high hydrophobicity and amphipathicity (hydrophobic moment) were observed to be correlated with increased haemolytic activity, while antimicrobial activity was found to be less dependent on these factors.

Structure–activity comparison was also performed on short 13-mer **g** and **h** compounds. Despite the short sequences, both peptides exhibited a helical structure. To increase the helicity of peptide **g**, which may be hampered by end-effects due to its short length, we replaced Leu21 with the helicogenic residue Aib. Short peptides containing Aib tend to adopt the 3_10_-helical conformation^17–19^, and, in fact, we could show, although the substitution did not have a significant effect on hydrophobicity, the presence of Aib induced the helix to adopt the prevailing 3_10_ conformation, as demonstrated by CD analysis and the NOESY spectrum^18–20^. Similar to what was observed above, the substitution of Leu21 increased helicity but again did not affect antimicrobial activity although it somewhat increased cytotoxicity against HaCaT cells (even remaining non-haemolytic, see Fig. 4). This result was unexpected and differs from the temporin-1DRa results obtained with the membranolytic frog skin peptides, in which Aib substitutions increased both its antimicrobial and cytolytic activities^34^.

We also tested the effects of elongating the acyl chain at the N-terminus of compound **b** (peptides **c**, **d**, and **e**) and a lipid chain insertion at the C-terminus (peptide **f**) of length similar to that of compound **d**. The lipopeptides showed a gradual decrease in helicity related to their lipid chain length compared with compound **b** under membrane-like conditions (SDS micelles). At the same time, all lipidated derivatives tended to aggregate. Moreover, the lipidated derivatives showed lower antimicrobial activity and increased cytotoxicity with respect to the compound **b**. However, in this respect, C-terminal lipidation was significantly less deleterious than N-terminal modification.

Lipidation and acylation have been shown to promote the antimicrobial activity of several AMPs^35, 36^ and to boost or modulate the antibacterial potential and the properties of already active AMPs^37^, also including some cathelicidins^38, 39^. However, this is not the case with our derivatives. The additional lipid chain has been shown to promote aggregation (see Supporting Information) even at low concentrations, and the acylated peptides are poorly active. The gradual decrease in helicity as a function of the length of the lipid tail length suggests that the alkyl chains interfere with helical structuration reducing the antimicrobial activity of the peptides. This hypothesis is also supported by the behaviour of peptide **f**, in which the addition of a hydrophobic tail at the C-terminus (Aun) did not affect helicity and partially affected its antibacterial potency. It resulted in higher antimicrobial activity and lower cytotoxicity than that of peptide **d** endowed with an acyl chain (lauric acid) of comparable length. These differences in activity are probably due to the higher helical content of compound **f** (82% versus 47%) compared to that of the related analogue **d**. However, in all cases, modification of compound **b** with lipid tails did not improve the biological activity of the peptide.

The proteolytic degradation of bioactive peptides shortens their in vivo lifespan and may alter their pharmacological profile^3^. The tests of resistance to the serine protease chymotrypsin presented here indicated that most PMAP-36 derivatives were susceptible to degradation and were hydrolysed by this protease within 10–20 min. The only exceptions were palmitoylated peptide **e** and Aib-containing peptide **h**, which were less than 50% degraded after 90 min.

The proteolytic fragment profile suggests that cleavage occurred at canonical sites^40^ between Leu14 and Lys15 and between Leu21 and Lys22. The introduction of Aib into the 13-mer compound **h** significantly reduced susceptibility to chymotrypsin. In the future, Aib or other non-proteinogenic residues could be introduced to obtain more stable peptides that retain antimicrobial activity and selectivity, ensuring that the peptides are not cytotoxic. As a preliminary assessment of the pharmacokinetic potential of the peptides, we also performed proteolytic degradation tests in serum for the most promising peptides **g** and **h**. Peptide **g** is rapidly degraded, as already observed in the presence of chymotrypsin. On the contrary, peptide **h** is capable of withstanding up to 180 min. Although this period is shorter than that observed in the presence of chymotrypsin, the result is still interesting, since the composition of the serum places the peptide in a more unfavourable environment (Supporting Information, Fig. S10). The observed resistance is reasonably attributable to the presence of the Aib residue. To obtain a more detailed profile of peptide stability, this preliminary study in serum needs to be extended to all peptide series.

In conclusion, we demonstrate that the α-helical cathelicidin PAMP-36 can be shortened to either side and that its central part (residues from 12 to 24) substantially retains its structural characteristics and antimicrobial activity against a wide spectrum of bacteria. We have also shown that the onset of a stable helical conformation is an essential prerequisite for the maintenance of antibacterial activity, but also that an additional increase in helicity and/or hydrophobicity does not improve the biological activities of peptides, as also indicated in the literature^33^. The derivatives **g** and **h,** which show the best ratio between activity/selectivity and peptide length, emerged as an interesting starting point for further optimisation to make them more resistant to proteolysis.

### Methods

#### Peptide synthesis

The solid phase peptide synthesis of the PMAP-36 analogues was performed on the Rink Amide MBHA resin, using standard Fmoc chemistry protocols^41, 42^. Deprotection of the Fmoc group was carried out with a 20% piperidine solution in *N*,*N*-dimethylformamide (2 × 10 min), and for the activation of the carboxylic groups, a mixture in a three-fold molar excess of amino acid and coupling reagent was used in the presence of a six-fold excess of DIPEA. Reaction time for each coupling was 50 min. On-resin Nα-acetylation was achieved using an Ac_2_O/DIPEA/DMF (1:0.5:20 ratio) mixture in DMF, reaction time 30 min. For the lipidated analogues, the introduction of the acyl chain was carried out on resin and required the pre-activation of octanoic acid, lauric acid and palmitic acid in the presence of HBTU, HOBT, and DIPEA. Fmoc-Aun-OH is commercially available (Iris Biotech GmbH, Marktredwitz, Germany) and requires the same activation protocol as the other Fmoc-amino acids.

At the end of the synthesis, each peptide was cleaved from the resin using a mixture of TFA, TIS, and water in a 95:2.5:2.5 ratio. The filtrates were collected and concentrated under a flow of nitrogen, and the crude peptide was precipitated by the addition of diethyl ether. The crude peptides were purified by flash chromatography on an Isolera Prime chromatographer (Biotage, Uppsala, Sweden) using a SNAP Cartridge KP-C18-HS 12g or preparative RP-HPLC on a Phenomenex C18 column (22.1 mm × 250 mm, 10 μm, 300 Å) using an Akta Pure GE Healthcare (Little Chalfont, U.K.) LC system equipped with an ultraviolet detector (flow rate of 15 mL/min) and a binary elution system: A, H_2_O; B, CH_3_CN/H_2_O [9:1 (v/v)]; gradient from 25 to 55% B in 30 min. The purified fractions were characterised by analytical HPLC–MS on a Phenomenex Kinetex XB-C18 column (4.6 mm × 100 mm, 3.5 μm, 100 Å) with an Agilent Technologies 1260 Infinity II HPLC system and a 6130 quadrupole LC/MS instrument. All compounds were > 95% pure.

The lyophilised peptides were then resuspended in DMSO (Sigma-Aldrich) and quantified using an Ultrospec 2100 pro spectrophotometer (Amersham Biosciences, Amersham, UK). Peptide concentrations were calculated spectrophotometrically using the absorbance at 280 nm and the molar extinction coefficients of tryptophan (ε = 5500/M cm). All peptides were stored frozen at – 20 °C.

#### Circular dichroism

The electronic CD curves in the far-UV region (below 260 nm) were obtained on a Jasco (Tokyo, Japan) model J-1500 spectropolarimeter with a fused quartz cell of 0.02 cm pathlength (Hellma, Mühlheim, Germany). The values are expressed in terms of [θ]_R_, the molar ellipticity per residue (deg × cm^2^/dmol). Spectra were recorded at room temperature in water, in spectrograde TFE (99.9% Acros Organics, Geel, Belgium) and 100 mM SDS solution at 3 × 10^−4^ M peptide concentration. The final molar peptide-to-lipid ratio is 1:300.

#### NMR

The monodimensional and correlated spectroscopy (COSY)/total correlation spectroscopy (TOCSY) and NOESY 2D NMR spectra of the selected analogues were obtained at 308 K by the use of a Bruker AVANCE NEO-600 spectrometer from an about 1.5 mM concentration sample dissolved in approximately 700 μL of TFE, d2 solution. Suppression of the solvent signal was achieved by the use of an excitation sculpting program^43^. All homonuclear spectra were acquired by collecting 512 experiments, each one consisting of 64–80 scans and 2 K data points. The spin systems of the coded amino acid residues were identified using standard double-quantum filtered-COSY^44^ and clean TOCSY^45^ spectra. In the latter case, the spin-lock pulse sequence was 70 ms long. NOESY experiments were utilized for sequence-specific assignments^26^. To avoid the problem of spin diffusion, the build-up curve of the volumes of the NOE cross peaks as a function of the mixing time (50–500 ms) was obtained first (data not shown). The mixing time of the NOESY experiment used for interproton distance determination was 150 ms (i.e., in the linear part of the NOE build-up curve).

#### Bacterial culture

The bacterial strains used *were Escherichia coli ATCC 25922, Staphylococcus aureus ATCC 25923, Klebsiella pneumoniae ATCC 700603, Acinetobacter baumannii ATCC 19606, Pseudomonas aeruginosa ATCC 27853 and Staphylococcus epidermidis ATCC 12228*. The reference bacterial strains used were purchased from American Type Culture Collection (ATCC) (Manassas, Virginia, USA). They were grown overnight (o/n) at 37 °C in complete Müller-Hinton broth (MHB, Difco) medium with shaking (130 rpm). The day after, the overnight bacterial cultures were diluted in fresh MHB (300 μL o/n culture in 10 mL) and incubated at 37 °C with shaking (130 rpm) for approximately 2 h (mid-log phase) until reaching an optical density (OD) at 600 nm (OD_600_) ≈ 0.3.

#### Minimum inhibitory concentration (MIC) assay

Peptides diluted in MHB to a concentration of 128 μM were added to the first column of wells of a 96-well round-bottom microtiter plate (Sarstedt, Milan, Italy) and serially diluted (1:2) in a final volume of 50 μL MHB was added to the next wells. Then, a mid-log bacterial cultures (OD _600_≈ 0.3) were diluted to 5 × 10^5^ colony-forming units (CFU)/mL in MHB and 50 μL of this bacterial suspension was added to each well. In this way, the final bacterial concentration was reduced to 2.5 × 10^5^ CFU/mL and the peptide concentration in the wells was also halved in a final volume of 100 μL (final bacteria load was 2.5 × 10^4^ CFU/well). The same amount of untreated bacteria in MHB was used as a control for bacterial growth. MHB (100 μL) was used as a negative control to check the sterility of the medium. The plate was sealed with parafilm to minimize evaporation and incubated overnight at 37 °C (approximately 18 h). MIC was determined by visual inspection at the end of incubation as the first clear well. Data are reported as moda of independent experiments repeated three times or four times in case of uncertain values.

#### Cytotoxicity assay on human cells

Cytotoxicity was measured by the MTT assay on human immortalized epidermal keratinocyte HaCaT cell line (DKFZ, Eppelheim, Germany). The HaCaT cells were grown in high-glucose Dulbecco’s modified Eagle’s medium (DMEM) (Sigma-Aldrich, Milan, Italy). DMEM medium was supplemented with 10% foetal bovine serum (FBS; Sigma, Milan, Italy), 2 mM glutamine (Sigma, Milan, Italy), 100 U/mL penicillin (Sigma, Milan, Italy) and 100 µg/mL streptomycin (Sigma, Milan, Italy). The cells were maintained at 37 °C in the presence of 5% CO_2_ and in a humidified incubator.

Cells were counted in a Burker-Türk chamber to prepare a suspension of 2 × 105 cells/ml in DMEM High Glucose Complete Medium (Sigma-Aldrich, Milan, Italy). 100 μL of this suspension was added to the wells of a 96-well flat-bottom transparent microtiter plate (Euroclone, Milan, Italy) specially treated for tissue culture. The plate was incubated for 24 h at 37 °C and 5% CO_2_ to allow the cells in each well to reach a semiconfluent state. The following day, serial dilutions (1:2) of the peptides, starting with 64 μM, were prepared in DMEM medium, and 100 μL of these solutions were used to replace the medium in the plate with HaCaT cells. The same amount of untreated DMEM high-glucose medium was used as a cell growth control. DMEM high-glucose complete medium (100 μL) was used as a negative control to check the sterility of the medium. The plate was sealed with Parafilm to minimize evaporation and incubated at 37 °C and 5% CO_2_ for 20 h. Then, 25 μL MTT (Sigma-Aldrich, Milan, Italy) dissolved in phosphate-buffered saline (PBS) was added to each well to a final concentration of 1 mg/mL MTT, and the plate was incubated for 4 h in the dark at 37 °C and 5% CO_2_.

At the end of incubation, 100 μL 10% (w/v) Igepal (Sigma-Aldrich, Milan, Italy) in 10 mM HCl was added to each well, and the plate was incubated overnight in the dark at 37 °C and 5% CO_2_. The absorbance of MTT at 570 nm was measured using a Nanoquant Infinite-M200Pro plate reader (Tecan, Männedorf, Switzerland). Student’s t-test was performed for all samples.

#### Haemolysis of ram red blood cell

The lytic potential towards eukaryotic cell membranes was assessed by measuring the release of haemoglobin from commercial ram red blood cells (RBCs) (Microbiol s.r.l., Cagliari, Italy) after exposure to the peptides. First, erythrocytes were isolated by centrifugation, washed with PBS and then suspended at 8% (v/v) in PBS. Secondly, 100 µL erythrocytes suspension was added into each well of a 96-well microplate. Then, 100 µL peptide solution was added to each well at different concentrations (the final concentration of erythrocytes suspension was 4% v/v), and the plates were incubated for 1 h at 37 °C and centrifuged at 1000 g for 10 min. Finally, 100 µL aliquots of supernatant were transferred to fresh 96-well microplate, and the release of haemoglobin was monitored by measuring the absorbance at 540 nm with Tecan microplate reader (Tecan Trading AG, Switzerland). 0% and 100% haemolysis were determined in PBS and 1% Triton X-100, respectively.

#### Proteolytic stability

The proteolytic stability of synthetic peptides was assessed using chymotrypsin (Sigma–Aldrich) and human serum (Sigma–Aldrich)^46^. The peptide (0.125 mg) was dissolved in the minimum amount (5 µl) of DMSO (dimethylsulfoxide) and then diluted (final volume: 50 µl) with Tris·HCl 50 mM, pH 7.8. Then, it was incubated with and without the enzyme solution (1.25 µg of enzyme in 150 µl buffer) at 37 °C for 24 h. After the workup procedures, the samples were analysed by RP-HPLC as described in the ‘peptide synthesis and characterization’ experimental section. A peptide not resistant to chymotripsin was used as a positive test. As a control, we used the 128-137 C-terminal portion of natural Endonuclease V. The peptide sequence is as follows: WYKYGKAIYA. All the degradation experiments were executed in triplicates.

#### Hydrophobicity

Peptide hydrophobicity was calculated as difference in retention times between peptide analogues **b–h** and peptide **a**. Each peptide was dissolved in MeOH to a concentration of 0.5 mg/mL and then subjected to RP-HPLC on an analytical Phenomenex Luna C18 (100 Å, 5µ) column using 0.1% TFA in 9:1 water:acetonitrile as solvent A, and 0.1% TFA in 9:1 acetonitrile:water as solvent B. The peptides were eluted using a linear gradient as follows: 5%B to 95%B over 30 min. The flow rate was 1 mL/min and the absorbance was measured at 215 nm and 280 nm. The retention time was recorded for each peptide.

### Supplementary Information

Supplementary Information.

## Data Availability

The datasets used and/or analysed during the current study are available from the corresponding author on reasonable request.
