# Supplementary material for: Structural and biological characterization of shortened derivatives of the cathelicidin PMAP-36
Source: Sci Rep. 2023 Sep 13;13:15132. doi: 10.1038/s41598-023-41945-1 (PMC10499915; doi:10.1038/s41598-023-41945-1)
Supplement: Supplementary file 1 — Supplementary Information. [file 41598_2023_41945_MOESM1_ESM.docx]

### SUPPORTING INFORMATION

### Structural and biological characterization of shortened derivatives of the cathelicidin PMAP-36

Barbara Biondi^1^, Luigi de Pascale^2^, Mario Mardirossian^2^, Adriana Di Stasi^2^, Matteo Favaro^3^, Marco Scocchi^2*^, and Cristina Peggion^1,3*^

^1^ Institute of Biomolecular Chemistry, Padova Unit, CNR, Padova, Italy

^2^ Department of Life Sciences, University of Trieste, Trieste, Italy

^3^ Department of Chemical Sciences, University of Padova, Padova, Italy

1. **NMR data of peptide b**

Table S1. ^1^H-NMR chemical shifts of peptide **b**.

1. **NMR data of peptide g**

Table S2. ^1^H-NMR chemical shifts of peptide **g**.

Figure S1. Figure S1. Fingerprint region of the NOESY spectrum (600 MHz) of peptide **g** in TFE solution (c= 1.2 mM, T=308 K)

Figure S2. Figure S2. Amide NH region of the NOESY spectrum (600 MHz) of peptide **g** in TFE solution (c= 1.2 mM, T=308 K)

1. **NMR data of peptide h**

Table S3. ^1^H-NMR chemical shifts of peptide **h**.

Figure S3. Amide NH region of the NOESY spectrum (600 MHz) of peptide **h** in TFE solution (c= 1.2 mM, T=308 K)

Figure S4. Fingerprint region of the NOESY spectrum (600 MHz) of peptide **h** in TFE solution (c= 1.2 mM, T=308 K). C^α^H_i_→NH_i+2_ and C^α^H_i_→NH_i+3_ cross-peaks are highlighted in red, and green, respectively.

Figure S5. Region of the NOESY spectrum (600 MHz) of peptide **h** in TFE solution (c= 1.2 mM, T=308 K). C^β^H_i_→NH_i+2_, C^β^H_i_→NH_i+3_ are highlighted in red and green respectively.

1. **Evaluation of solubility and aggregation**

Figure S6. Solubility of PMAP-36 derivatives at different concentrations in PBS after 4h and 24 h incubation

Figure S7. Solubility of PMAP-36 derivatives at different concentrations in completed Mueller-Hinton broth after 4h and 24 h incubation

1. **Peptide stability**

Figure S8. HPLC profile with labeled peaks corresponding to the hydrolysis products for peptide **d**.

Figure S9. HPLC profile for the proteolytic degradation of the control peptide.

Figure S10. Proteolytic resistance of peptide g and h in the presence of chymotrypsin or serum.

1. **Peptide selectivity**

Table S4. Selectivity index (SI) of the tested peptides for each bacterial species based on cytotoxicity.

Table S5. Selectivity index (SI) of the tested peptides for each bacterial species based on haemolysis.

1. **NMR data of peptide b**

Table S1. ^1^H-NMR chemical shifts of peptide **b**.

| Residue | NH | α | β | other |
| --- | --- | --- | --- | --- |
| Ac^0^ |  |  |  | 2.14 |
| Lys^12^ | 7.66 | 4.15 | 1.95 | γ 1.66; δ 1.86; ε - |
| Arg^13^ | 8.02 | 4.20 | 2.07, 2.00 | γ 1.88, 1.82; δ 3.29; NHε 7.21 |
| Leu^14^ | 7.36 | 4.25 | - | γ -; δ 1.05, 0.97 |
| Lys^15^ | 7.72 | 4.20 | 2.02 | γ 1.70, 1.58; δ 1.85; ε 3.08; NHε 7.43 |
| Lys^16^ | 8.21 | 3.92 | - | γ -; δ -; ε - |
| Ile^17^ | 8.58 | 3.79 | - | γ - |
| Gly^18^ | 8.11 | 4.03, 3.87 |  |  |
| Lys^19^ | 7.77 | 4.22 | 2.04 | γ 1.66, 1.56; ε 3.06; NHε 7.39 |
| Val^20^ | 7.96 | 3.92 | 2.01 | γ 1.05 |
| Leu^21^ | 7.72 | 4.20 | 1.85 | - |
| Lys^22^ | - | - | - | *ND for overlapping* |
| Trp^23^ | 8.07 | 4.57 | 3.67, 3.50 | H1 9.16; H2 7.43; H4 7.7; H5 7.15; H6 7.24; H7 7.48 |
| Ile^24^ | 8.61 | 3.71 | 2.11 | γ 1.37; γ(CH_3_) 1.10; δ 1.02 |
| Ala^25^ | 8.27 | 4.09 | 1.57 |  |
| Lys^26^ | 7.77 | 4.10 | - | - |
| Ile^27^ | 8.47 | 4.23 | 1.97 | γ 1.62; γ(CH_3_) 1.15; δ 0.97 |
| Val^28^ | 7.89 | 3.91 | 2.40 | γ 1.16 |
| Gly^29^ | 8.08 | 3.79 |  |  |
| Ser^30^ | 7.79 | 4.55 | 4.12, 3.99 |  |
| Ile^31^ | 7.62 | 4.28 | 2.02 | γ 1.64, 1.30; γ(CH_3_) 1.03; δ 0.94 |
| -NH_2_ |  |  |  | 7.25, 6.14 |

1. **NMR data of peptide g**

Table S2. ^1^H-NMR chemical shifts of peptide **g**.

| Residue | NH | α | β | other |
| --- | --- | --- | --- | --- |
| Ac^0^ |  |  |  | 2.15 |
| Lys^12^ | 7.98 | 4.08 | 1.88, 1.79 | γ 1.52; δ 1.64; ε 3.05 |
| Arg^13^ | 7.93 | 4.12 | 1.98, 1.93 | γ 1.78; δ 3.26; NHε 7.00 |
| Leu^14^ | 7.31 | 4.25 | 1.87, 1.72 | γ 1.67; δ 1.03, 0.95 |
| Lys^15^ | 7.78 | 4.18 | 1.99 | γ 1.61, 1.50; δ 1.77; ε 3.05 |
| Lys^16^ | 7.62 | 4.16 | 1.93, 1.86 | γ 1.38; δ 1.67; ε 3.05 |
| Ile^17^ | 7.92 | 3.93 | - | γ 1.01 |
| Gly^18^ | 8.17 | 3.86 |  |  |
| Lys^19^ | 7.71 | 4.15 | 1.98 | γ 1.52; δ 1.79, 1.67; ε 3.06 |
| Val^20^ | 7.49 | 4.21 | 1.92 | γ 0.96, 0.91 |
| Leu^21^ | 7.83 | 3.93 | 2.30 | γ 1.52; δ 1.12, 1.06 |
| Lys^22^ | 8.10 | 4.25 | 1.81 | γ 1.50; δ -; ε 3.06 |
| Trp^23^ | 7.78 | 4.73 | 3.14 | H1 9.10; H2 7.21; H4 7.18; H5 7.70; H6 7.24; H7 7.46 |
| Ile^24^ | 7.49 | 4.20 | 1.93 | γ 1.21 |
| -NH_2_ |  |  |  | 6.89 |

Figure S1. Fingerprint region of the NOESY spectrum (600 MHz) of peptide **g** in TFE solution (c= 1.2 mM, T=308 K)

Figure S2. Amide NH region of the NOESY spectrum (600 MHz) of peptide **g** in TFE solution (c= 1.2 mM, T=308 K)


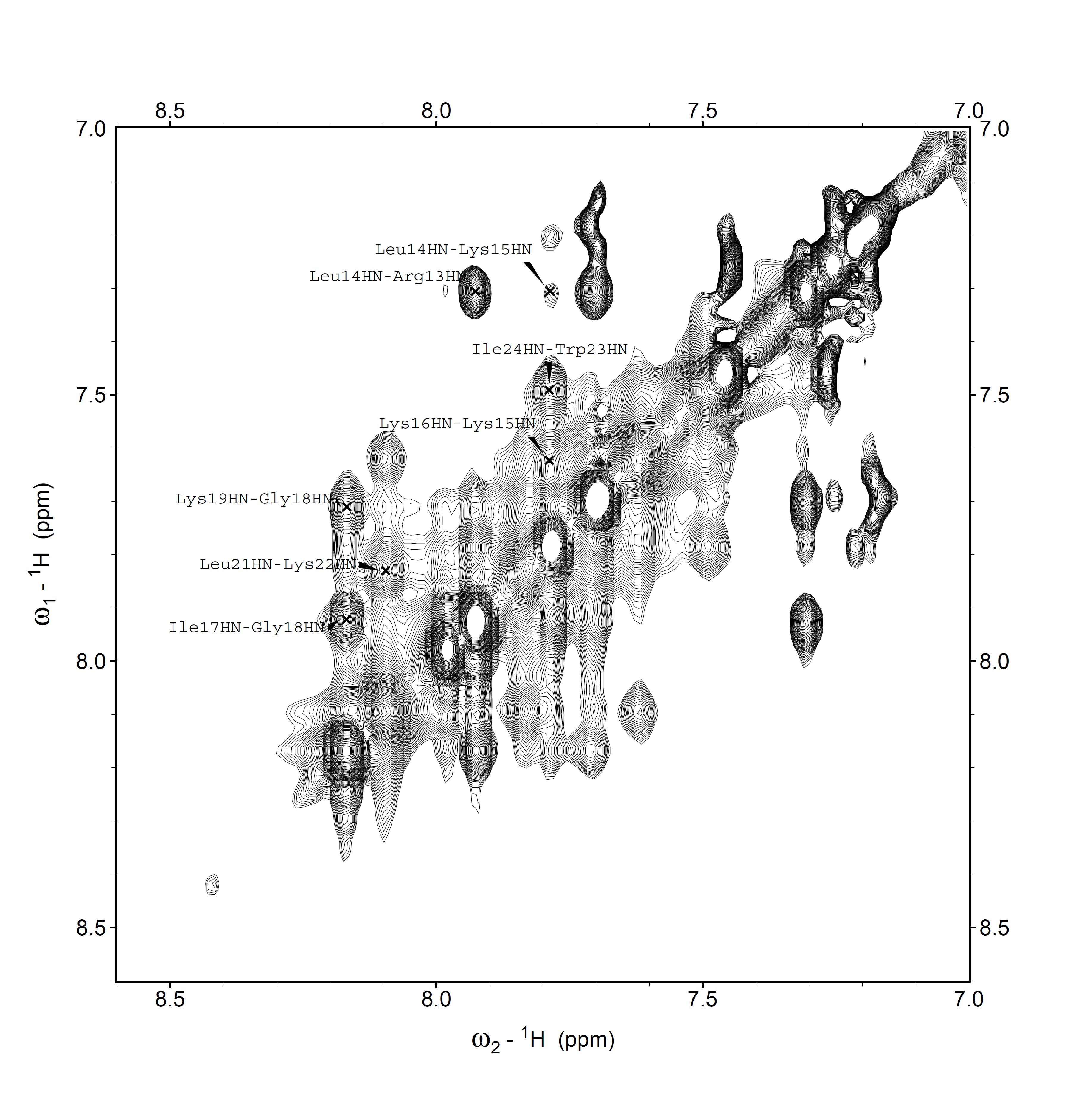


1. **NMR data of peptide h**

Table S3. ^1^H-NMR chemical shifts of peptide **h**.

| Residue | NH | α | β | other |
| --- | --- | --- | --- | --- |
| Ac^0^ |  |  |  | 2.14 |
| Lys^12^ | 8.01 | 4.07 | 1.88 | γ 1.53; δ 1.64; ε 3.05 |
| Arg^13^ | 7.34 | 4.25 | 1.86 | γ 1.69; δ 3.07; NHε 7.31 |
| Leu^14^ | 7.95 | 4.12 | 1.94 | γ 1.77; δ - |
| Lys^15^ | 7.65 | 4.28 | 1.98 | γ 1.51; δ 1.67, 1.77; ε 3.05 |
| Lys^16^ | 7.76 | 4.19 | 1.98 | γ 1.60, 1.50; δ 1.77; ε 3.04 |
| Ile^17^ | 7.85 | 3.97 | 1.98 | γ 1.02 |
| Gly^18^ | 8.08 | 3.91 |  |  |
| Lys^19^ | 7.25 | 4.16 | 1.81 | γ 1.33; δ 1.65; ε 3.38; NHε 7.10 |
| Val^20^ | 7.68 | 9.91 | 2.25 | γ 1.11, 1.06 |
| Aib^21^ | 8.07 | - | 1.54, 1.48 |  |
| Lys^22^ | 7.66 | 4.16 | 1.95 | γ 1.67, 1.51; δ 1.77; ε 3.06; NHε 7.03 |
| Trp^23^ | 7.94 | 4.76 | 3.45, 3.38 | H1 9.11; H2 7.25; H4 7.13; H5 7.72; H6 7.25; H7 7.45 |
| Ile^24^ | 7.51 | 4.25 | 1.97 | γ 1.25; γ(CH_3_) 0.97; δ 0.91 |
| -NH_2_ |  |  |  | 7.00, 6.00 |

Figure S3. Amide NH region of the NOESY spectrum (600 MHz) of peptide **h** in TFE solution (c= 1.2 mM, T=308 K)


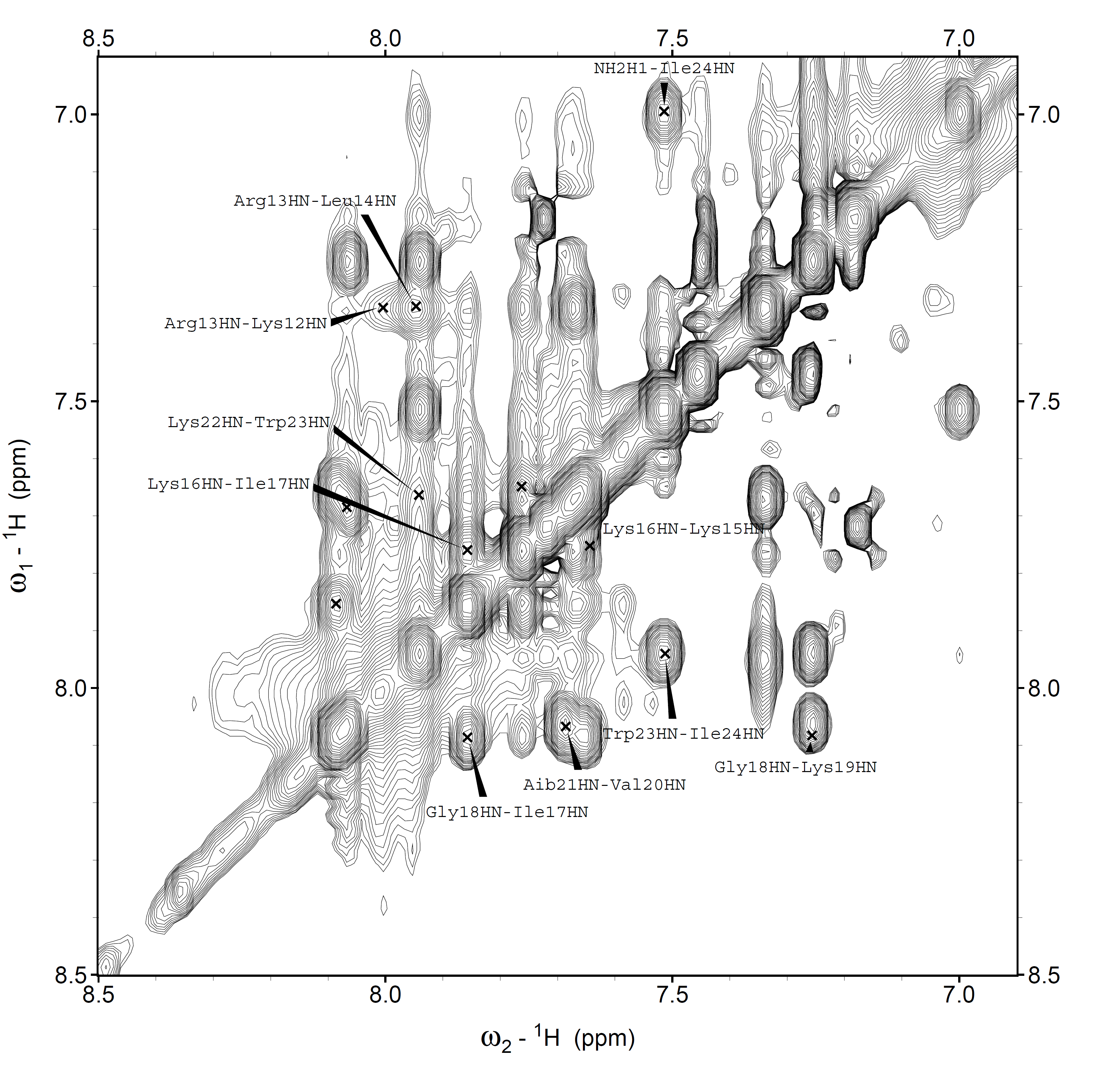


Figure S4. Fingerprint region of the NOESY spectrum (600 MHz) of peptide **h** in TFE solution (c= 1.2 mM, T=308 K). C^α^H_i_→NH_i+2_ and C^α^H_i_→NH_i+3_ cross-peaks are highlighted in red, and green, respectively.

Figure S5. Region of the NOESY spectrum (600 MHz) of peptide **h** in TFE solution (c= 1.2 mM, T=308 K). C^β^H_i_→NH_i+2_, C^β^H_i_→NH_i+3_ are highlighted in red and green respectively.

**4. Evaluation of solubility and aggregation**


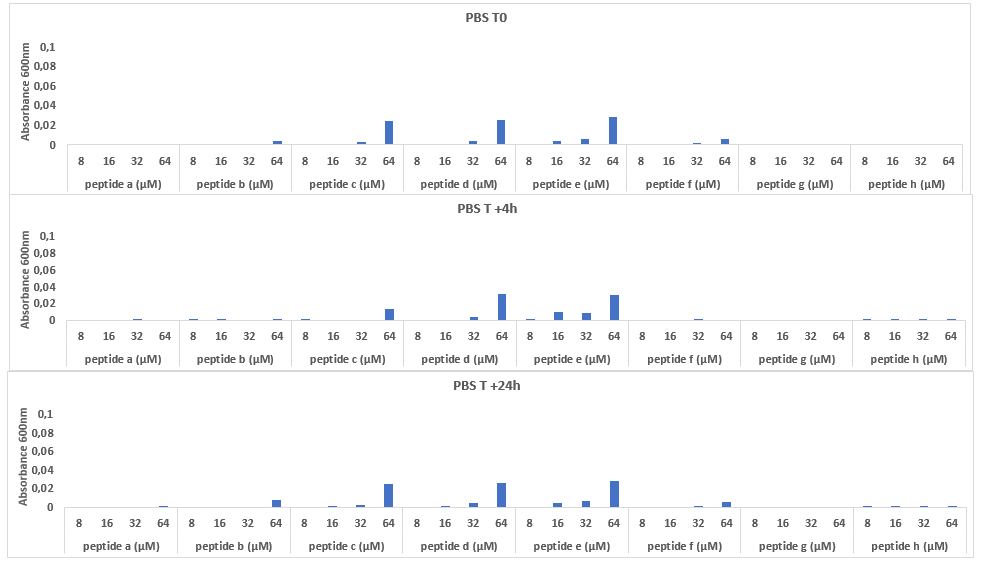


Figure S6 Solubility of PMAP-36 derivatives at different concentrations in PBS after 4h and 24 h incubation


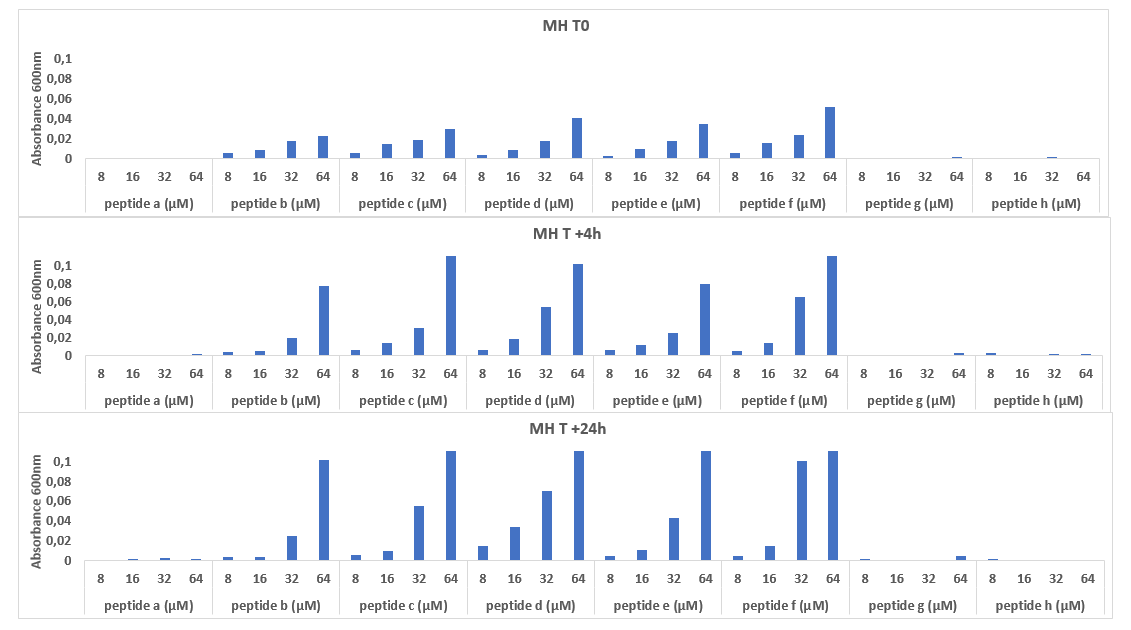


Figure S7 Solubility of PMAP-36 derivatives at different concentrations in completed Mueller-Hinton broth after 4h and 24 h incubation

**5. Peptide stability**


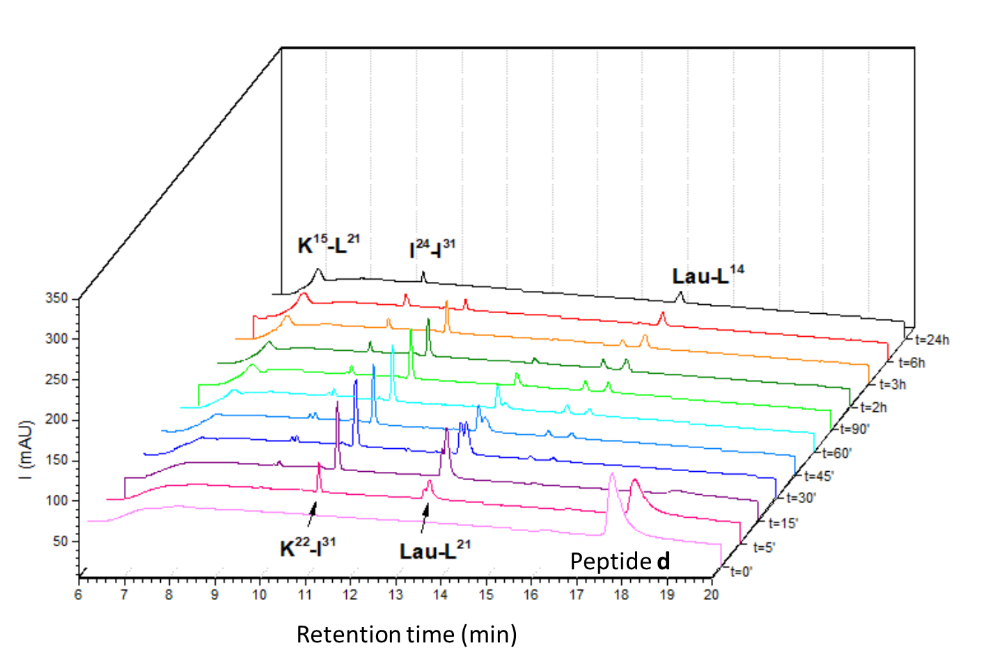


Figure S8. HPLC profile with labeled peaks corresponding to the hydrolysis products for peptide **d**.


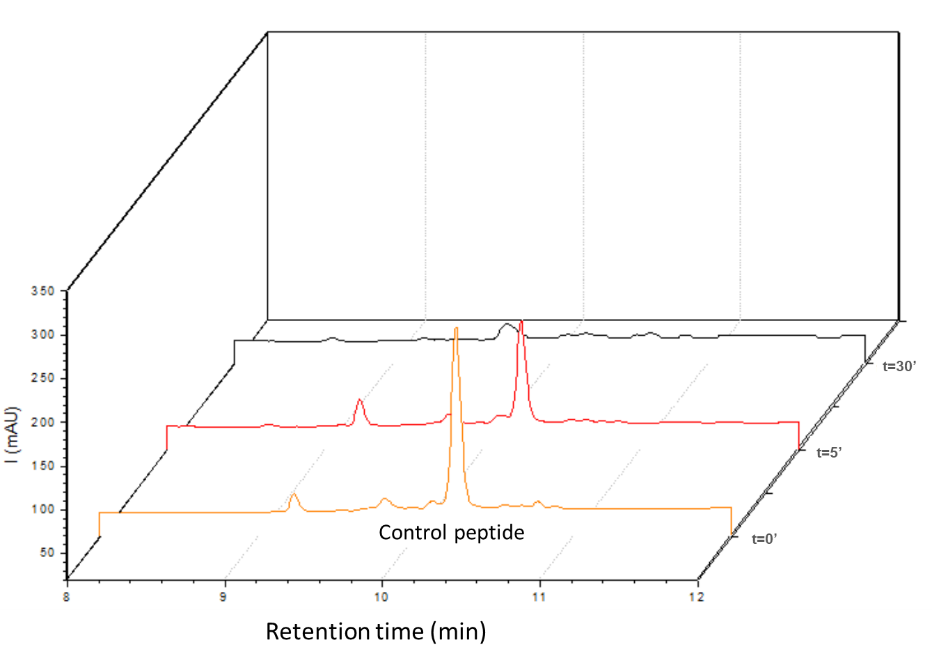


Figure S9. HPLC profile for the proteolytic degradation of the control peptide.

Figure S10. Proteolytic resistance of peptide **g** and **h** in the presence of chymotrypsin (blue) or serum (green). The bars indicate the final time when the HPLC-MS signal of the peptides is still detectable in solution.

**6. Peptide selectivity**

Table S4. Selectivity index (SI) of the tested peptides for each bacterial species based on cytotoxicity

SI are reported as ratio between cytotoxicity and MIC

* The IC50 was considered as the peptide concentration at which cell viability is reduced by 50% compared to the untreated control.

n.a. indicates conditions where both numbers were not available but there was only the indication > than 32 µM or 64 µM.

Table S5. Selectivity index (SI) of the tested peptides for each bacterial species based on haemolysis.

SI of the tested peptides for each bacterial species are reported as ratio between minimal haemolysis concentration (MHC) and MIC.

**MHC was taken as the lowest concentration of peptides which induced 10% of haemolysis of RBC.

n.a. indicates conditions where both numbers were not available but there was only the indication > than 32 µM or 64 µM.
